# Enrichment of H3S28p and H3K9me2 Epigenetic Marks on Inflammatory-Associated Gene Promoters in Response to Severe Burn Injury

**DOI:** 10.3390/life14121581

**Published:** 2024-12-01

**Authors:** Osvaldo Arias-Pérez, Thelma Escobedo-Tapia, Cecilia Cintora-Ahumada, Lizbel León-Solís, Norberto Leyva-García, Elena Aréchaga-Ocampo, Rafael Franco-Cendejas, Oscar Hernández-Hernández, Rocío Suárez-Sánchez

**Affiliations:** 1 Laboratorio de Medicina Genómica, Departamento de Genética, Instituto Nacional de Rehabilitación “Luis Guillermo Ibarra Ibarra”, Mexico City 14389, Mexico; 2 Departamento de Ciencias Naturales, Universidad Autónoma Metropolitana-Unidad Cuajimalpa, Mexico City 05348, Mexico; 3 Departamento de Microbiología, Escuela Nacional de Ciencias Biológicas, Instituto Politécnico Nacional (IPN), Mexico City 11340, Mexico; 4 Subdirección de Investigación Biomédica, Instituto Nacional de Rehabilitación “Luis Guillermo Ibarra Ibarra”, Mexico City 14389, Mexico

**Keywords:** burn injury, histone H3, posttranslational modification, inflammation, sepsis

## Abstract

Background: Severe burns activate systemic inflammation and lead to an increase in cytokine levels. Epigenetic elements are key regulators of inflammation; however, their involvement in severe burns has not been studied. In this work, we aimed to unveil the histone H3 posttranslational modifications (PTM) profile and their enrichment in promoters of inflammatory genes in response to severe burns. Methods: The levels of H3 PTMs were analyzed by ELISA assays in circulating cells from burn patients. ChIP assays were conducted to evaluate the enrichment of H3K9me2 and H3S28p at the promoter of CXCL8, IL-17, TNFA, IL-6, FOS, and IL-1B genes. Results: We found that eight H3 PTMs decreased at 5 days post-burn. Burn patients showed a decreased enrichment of H3K9me2 in CXCL8, IL-17, and TNFA promoters, whereas IL-6, FOS, and IL-1B promoters displayed an H3S28p enrichment diminution during the first 10 days post-burn. Interestingly, burn-injured septic patients exhibited an increased enrichment of H3K9me2 in TNFA, IL-1B, CXCL8, and IL-17 promoters, whereas H3S28p was increased in promoters of TNFA and IL-1B at 1 dpb. Conclusion: Severe burns trigger epigenetic changes and differential H3 PTM enrichment at inflammation gene promoters. Epigenetic misregulation of H3 may be involved in sepsis occurrence after severe burn injury.

## 1. Introduction

Burns are tissue injuries caused by heat, electricity, friction, radiation, or chemicals. According to the World Health Organization (WHO), burns are a global public health problem, accounting for an estimated 180,000 deaths annually [1]. The incidence of burns in Mexico is approximately 3700 patients per 100,000 inhabitants. In 2013, the National Epidemiological Surveillance System reported 126,786 new cases and 627 burn-related deaths were confirmed by the National Institute of Statistics and Geography (INEGI) in 2018. At the end of 2022, 60,335 new cases had been confirmed [2,3,4].

Burn injury induces local changes characterized by the activation of inflammatory pathways and the release of several cytokines by immune effector cells [5,6,7]. When the burn is severe, it causes significant damage to the skin and underlying fat, muscle, and even bone tissue, triggering a systemic response characterized by metabolic, respiratory, and inflammatory alterations that, in some cases, may result in sepsis, multiple organ failure, and death [8,9,10,11,12]. The percentage of total body surface area (%TBSA) injured, the depth and type of burn, the specific body parts involved, and the age of the patient determine the severity of the burn. When >10% TBSA in children or older people, >20% TBSA in adults, >5% full thickness, high-voltage electrical burns, and significant burns to the genitalia and face occur, the burn is classified as severe [11,13,14]. The systemic inflammation unchained by severe burns is characterized by a generalized rise in the expression of inflammation-related cytokines, including interleukin (IL)-1β, IL-6, IL-8, IL-17, tumor necrosis factor α (TNF-α) (proinflammatory), and IL-2, IL-4, and IL-10 (anti-inflammatory) [7,15,16]. Cytokine levels must be precisely controlled; a misregulation is associated with the risk of sepsis and not survival, thus having a crucial impact on the outcome of burn patients [5,15,17,18,19,20,21,22].

Epigenetic factors have been described as key regulators of inflammatory processes in several pathologies [23,24,25,26,27,28,29,30,31,32,33]; however, their role remains poorly understood in burn injuries. In animal models, alterations in DNA methylation and posttranslational modifications (PTMs) of histones have been described after burn injuries [34,35,36,37,38,39,40]. Recently, it has been reported in murine burn models that PTM variants of histone H3 are misregulated in response to burn injury, levels of H3K9ac were found to decrease in intestinal mucosal epithelial cells of scalded rats [37], and levels of H3K4me1 and H3K4me3 were increased in the spinal cord of burn-injured mice [39]. In burn patients, the upregulation of the long intergenic non-coding RNA 00174 (linc00174) promotes angiogenesis during recovery [41]. In addition, neutrophil activation and NETosis, which have a key role in burn injury progression, were associated with elevated serum levels of citrullinated histone H3 [42]. Although the involvement of epigenetic factors in the pathophysiology of burn injury has been explored, the potential contribution that H3 PTMs could have in systemic response has not been addressed in the case of severe burns.

In this work, we describe for the first time an altered profile of epigenetic marks in response to severe burn injury, including the methylation, phosphorylation, and acetylation of histone H3. By studying the enrichment of histone modification in promoters of inflammatory-related genes, we propose a potential involvement of H3S28p and H3K9me2 in the control and modulation of cytokine levels in response to severe burn injury.

## 2. Materials and Methods

### 2.1. Human Subjects and Sample Collection

Twelve male patients > 18 years who were admitted to the burn intensive care unit within 48 h post burn and presented a percentage of total body surface area (TBSA) > 15% were included in this study (Table 1). Among these, 7 patients were victims of fire burn and 5 of electrical burn injury. Six healthy male volunteers without any inflammatory conditions and who displayed an unremarkable medical history were included. The Research Ethics Committee of the National Institute of Rehabilitation approved this work, and written informed consent was obtained from all participants. Blood samples were collected from healthy volunteers and burn patients on day 1 post-burn (1 dpb), 5 days post-burn (5 dpb), and 10 days post-burn (10 dpb). The samples were processed within 2 h following collection. Plasma was obtained by centrifugation at 1800 g for 20 min. Peripheral blood mononuclear cells (PBMCs) were isolated using Lymphoprep (Axis Shield, Dundee, UK) by centrifugation at 800 g with brake-off for 30 min. Residual red blood cells were lysed with RBC lysis buffer (Qiagen, Valencia, CA, USA).

### 2.2. IL-6 Quantification

Interleukin 6 levels in plasma were evaluated by a Human IL-6 Elisa Kit (RAB0306, Sigma-Aldrich, St. Louis, MO, USA) according to the manufacturer’s instructions. Briefly, 100 µL of diluted plasma or standard controls were added to each well. Covered plates were incubated at room temperature for 2.5 h. After four washes, 100 µL of Biotinylated Detection Antibody was added to each well, incubated for 60 min at room temperature with gentle shaking, and washed. Additionally, 100 µL of HRP-Streptavidin Solution was added to each well and incubated for 45 min at room temperature with gentle shaking. Washes were repeated. For signal detection, 100 µL of Elisa Colorimetric TMB Reagent was added and incubated away from light for 30 min. Finally, 50 µL of Stop Solution was added, and the absorbance was read at 450 nm immediately.

### 2.3. Histone Extraction

PBMCs were processed by the Histone Extraction Kit (ab113476, Abcam, Cambridge, UK) according to the manufacturer’s recommendations. Briefly, cells were re-suspended in pre-lysis buffer at 10^7^ cells/mL and incubated on ice for 10 min with gentle stirring. Cells were centrifuged at 10,000 rpm for 1 min at 4 °C, and the supernatant was removed. Cells were re-suspended in lysis buffer and incubated on ice for 30 min. After centrifugation at 12,000 rpm for 5 min, the supernatant was recovered and Balance-DTT Buffer was added. Histone proteins were quantified by a DC Protein Assay (Bio-Rad, Hercules, CA, USA).

### 2.4. Histone Post-Translational Modification Assay

Twenty-one PTMs of histone H3 were evaluated by using the Histone H3 Modification Assay Kit (ab185910, Abcam, Cambridge, UK). All procedures were performed according to the manufacturer’s instructions. Briefly, 49 µL of antibody buffer was added to each well, including blank wells and standard controls (5 and 25 ng of Control proteins/well). Then, 100 ng of each histone sample was added per well to reach 50 uL total volume, and the plate was incubated at 37 °C for 2 h. After that, three washes with Wash Buffer were performed. Then, 50 µL of capture antibody was added and incubated for 60 min at room temperature. For signal detection, 100 µL of developer solution was added and incubated away from light for 10 min. Finally, 100 µL of stop solution was added, and the absorbance was read within 2 to 10 min at 450 nm with a reference wavelength of 655 nm.

### 2.5. Confirmation of H3K4me3, H3K9me2, H3K27me1, and H3S28p Levels

By using independent ELISA assays, we confirmed the levels of H3K4me3, H3K9me2, H3K27me1, and H3S28p (ab115056, ab115062, ab115068, and ab115129, respectively, Abcam, Cambridge, UK) in the chromatin of the PMBCs according to the instructions of the manufacturer. Briefly, 50 µL of antibody buffer was added to each well, including blank wells and standard controls (1, 3, 7, 15, and 60 ng). Then, 1 µg of each histone sample was added per well. Covered plates were incubated at room temperature for 2 h. After that, three washes with Wash Buffer were performed. Then, 50 µL of detection antibody was added and incubated for 60 min at room temperature on an orbital shaker and washed again. For signal detection, 100 µL of color developer was added and incubated away from light for 10 min, until the change of color was detected. Finally, 50 µL of Stop Solution was added, and the absorbance was read within 2 to 15 min at 450 nm. According to the manufacturer, the quantification of PTMs was calculated as the relative level of burn samples over control samples.

### 2.6. Chromatin Immunoprecipitation (ChIP)

For ChIP assays, PBMCs were crosslinked for 10 min with 1% formaldehyde at room temperature. The reaction was stopped by adding glycine to 125 mM of the final concentration, and cells were pelleted by centrifugation. Cells were resuspended in a cell lysis buffer (10 mM Tris-HCl pH 7.5, 10 mM NaCl, and 0.5% NP40) and incubated for 10 min on ice. The nuclear pellet was obtained by centrifugation and resuspended in a nuclear lysis buffer (50 mM Tris-HCl pH 7.5, 10 mM EDTA, and 1% SDS). Nuclear extract was sonicated at 10% amplitude for 10 min in a 705 Sonic Dismembrator (Thermo Fisher Scientific. Waltham, MA, USA). The soluble fraction was diluted 2.5-fold with dilution buffer (16.7 mM Tris-HCl pH 7.5, 167 mM NaCl, 1.2 mM EDTA pH 8.0, and 1.1% triton X-100). Additionally, 10 µg of chromatin was used for each immunoprecipitation with 1 µg of anti-H3K27me1 antibody (ab194688, Abcam, Cambridge, UK) or an irrelevant antibody (IgG). The chromatin–antibody mixture was incubated overnight at 4 °C with gentle agitation. The immunoprecipitated complexes were recovered by incubation with protein A agarose for 2 h. The immunoprecipitated material was washed with a low salt buffer (20 mM Tris-HCl pH 8, 2 mM de EDTA, 1% triton X-100, 0.1% SDS, and 150 mM NaCl), then with a high NaCl buffer (20 mM Tris-HCl pH 8, 2 mM de EDTA, 1% triton X-100, 0.1% SDS, and 500 mM NaCl), and finally with a high LiCl buffer (20 mM Tris-HCl pH 8, 2 mM de EDTA, 1% triton X-100, 0.1% SDS, and 500 mM LiCl). The immunoprecipitated fraction was eluted by incubating with an elution buffer (25 mM Tris-HCl pH 8, 5 mM EDTA, and 0.5% SDS) for 15 min at 65 °C. Samples were descrosslinked by adding 0.2 mg/mL proteinase K and incubated for 2 h at 42 °C, followed by an overnight incubation at 65 °C. Immunoprecipitated DNA was purified, and real-time PCR (qPCR) reactions for promotor regions of selected genes were conducted. Significance was tested against values corresponding to healthy controls; DNA fragments obtained with normal rabbit IgG were used as negative controls (Supplementary Figure S1 shows background IgG analysis). The sequence of primers used in this study were: CXCL8 (Fw 5′-CAGAGACAGCAGAGCACAC-3′, Rv 5′-ACGGCCAGCTTGGAAGTC-3′), IL-17 (Fw 5′-TCACATGATGCTGTGCAGTAAGAA-3′, Rv 5′-TGGTACGAGATAATGGAAACAAAG-3′), IL-1b (Fw 5′-CGTGGGAAAATCCAGTATTTTAATG-3′, Rv 5′-CAAATGTATCACCATGCAAATATGC-3′), TNFA (Fw 5′-AACCGAGACAGAAGGTGCAG-3′, Rv 5′-TGTGCCAACAACTGCCTTTA-3′), IL-6 (Fw 5′-TCGTGCATGACTTCAGCTTT-3′, Rv 5′-GCGCTAAGAAGCAGAACCAC-3′), and FOS (Fw 5′-GAGCAGTTCCCGTCAATCC-3′, Rv 5′-GCATTTCGCAGTTCCTGTCT-3′).

### 2.7. Statistical Analysis

Descriptive data of patients were expressed as mean ± standard error of the mean (SEM) or as a range of corresponding parameters. Age and IL-6 level data of control and patients were analyzed using the Mann–Whitney and Kruskal–Wallis tests, respectively. A comparison of total and PTM levels of histone H3 between control and burn patients was performed using an unpaired *t*-test. A comparison of histone H3 PTMs data of controls and patients at 1, 5, and 10 dpb was made by using one-way ANOVA and Dunnet’s multiple comparison test. ChIP data analysis was performed with a one-way ANOVA and Bonferroni multiple comparison test. The dynamic of inflammation gene promoter occupancy was evaluated by using a two-way ANOVA with Bonferroni multiple comparison test. All analyses were performed by using GraphPad Prism 10.3.1 (GraphPad Software; Boston, MA, USA). A *p*-value < 0.05 was considered statistically significant.

## 3. Results

### 3.1. Demographic and Clinical Characteristics of Burn Patients

The demographics of the patients and control subjects are summarized in Table 1. The burn patients had a mean age of 30.83 years (15–81 years), and the healthy controls of 33.67 years (15–71 years), with no statistical differences (*p* = 0.7971). The injury of seven of the burn patients was caused by fire, and by electricity for the other five. The mean extent of the burn was 36.92% TBSA, ranging from 15 to 60%. In order to confirm the inflammatory state in burn patients, we evaluated plasma levels of IL-6, a well-recognized inflammatory cytokine that increases after severe burn response [15]. Exacerbated plasma IL-6 levels at 1, 5, and 10 dpb confirmed the inflammatory state of burn patients. The plasma concentration of IL-6 was < 3 pg/mL in controls, as expected (Table 1).

### 3.2. Levels of Histone H3 Post-Translational Modifications Are Altered in Response to Severe Burn Injury

**Figure 1:**
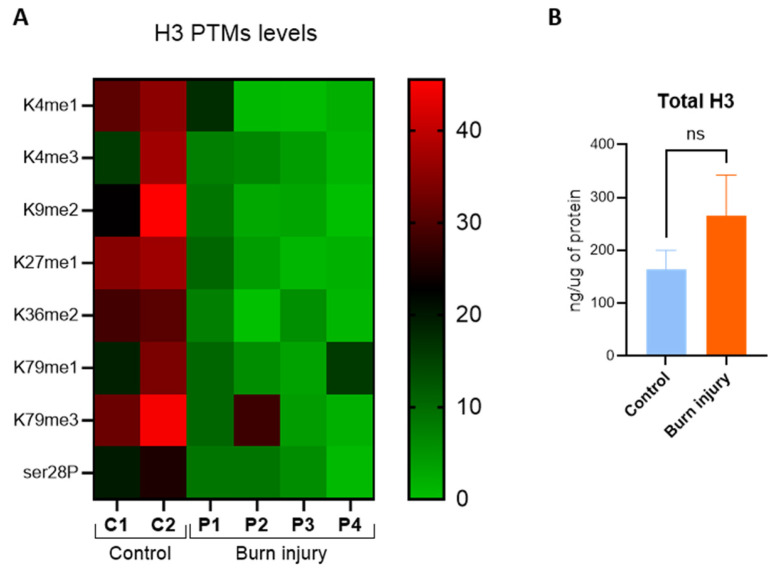
Levels of post-translational modifications (PTMs) of histone H3 in burn patients. (**A**) Protein extracts from PMBCs of 4 burn patients and 2 healthy volunteers were analyzed by a Histone H3 Modification Assay Kit at 5 days post-burn. The heat map shows decreased levels (ng/ug of protein) of 8 PTMs of histone H3 in burn patients compared to controls. We obtained results of *p* < 0.05 by using a multiple unpaired *t*-test. (**B**) Quantification of total histone H3 (ng/ug of protein) in leukocytes of severely burned patients at 5 days post-burn. Mean ± SEM, ns = non-significant by using unpaired *t*-test.

To study the changes in PTMs of histone H3 in response to severe burn injury, we initially analyzed PMBCs from burn patients at 5 dpb. By using a multiplex ELISA array, we evaluated 21 PTMs, including 15 methylations, 4 acetylations, and 2 phosphorylations (Supplementary Figure S2). Interestingly, a decrease in the levels of eight PTMs, including H3K27me1, H3K4me3, H3S28p, and H3K9me2, was observed in burned patients compared to controls, with no significant changes in the total H3 levels (Figure 1A,B). The alteration of both activation marks, H3K27me1 [43,44], H3K4me3 [26,45], and H3S28p [46,47], and repression marks, H3K27me3 [48] and H3K9me2 [49,50], suggests the involvement of epigenetic regulation in the inflammatory response caused by burn injury.

**Figure 2:**
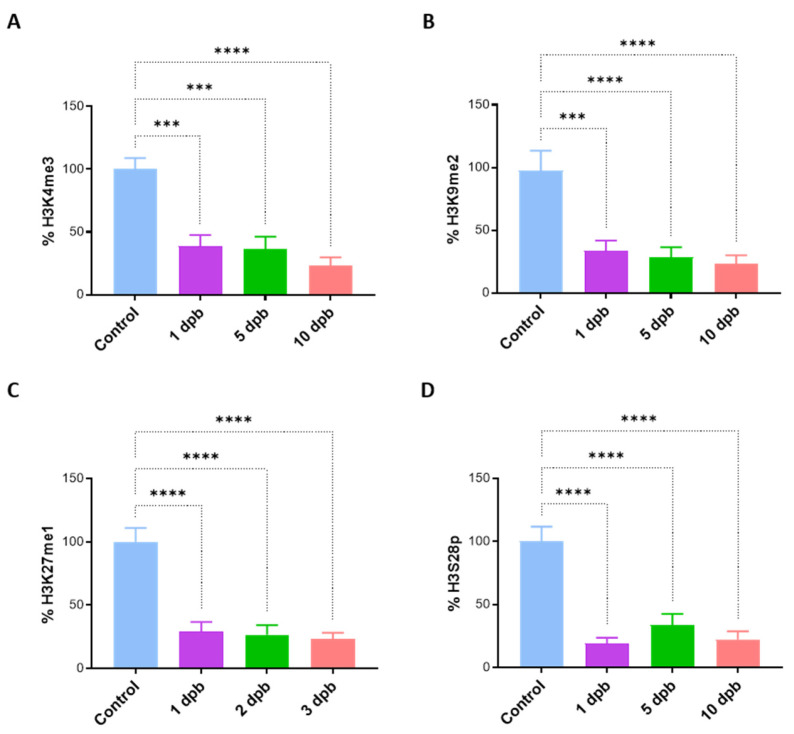
Levels of histone H3 PTMs in burn patients are decreased and sustained throughout the first 10 days post-burn. (**A**) H3K4me3, (**B**) H3K9me2, (**C**) H3K27me1, and (**D**) H3S28p levels in PMBCs of 6 controls and 12 severe burn patients were quantified at 1, 5, and 10 days post-burn. Bar graphic shows mean ± SEM, *** *p* < 0.001, **** *p* < 0.0001 by using one-way ANOVA and Dunnet’s multiple comparison test.

In order to deepen the study of PTM alterations, we decided to confirm the levels of H3K4me3, H3K9me2, H3K27me1, and H3S28p in PMBCs of 6 healthy controls and 12 severely burned patients at 1, 5, and 10 dpb. H3K4me3 showed a statistically significant decrease in burn patients, around 50% of the level in healthy controls at 1 dpb, which was sustained throughout all the studied times (Figure 2A). Likewise, H3K9me2 and H3K27me1 decreased by more than 50% in burn patients in all the studied times (Figure 2B,C). H3S28p was the most affected mark, with levels at 1 and 10 dpb around 25% of those displayed in healthy controls, and 50% at 5 dpb (Figure 2D). These assays revealed that PTM changes initiate as early as 1 dpb and are sustained for at least the first 10 days after the burn.

### 3.3. Recruitment of H3K9me2 and H3S28p in Inflammatory Gene Promoters Is Altered in Severe Burn Injury

**Figure 3:**
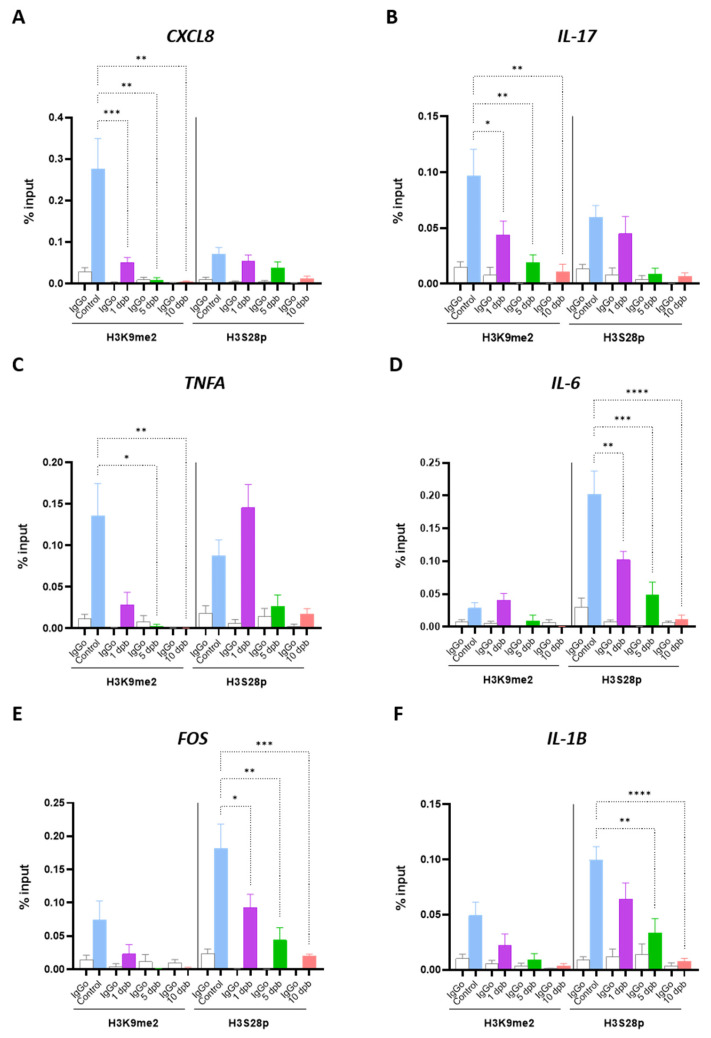
Altered recruitment of epigenetics marks in inflammatory gene promoters in response to burn injury. Chromatin immunoprecipitation assays were performed in PMBCs of control and burn patients at 1, 5, and 10 dpb to evaluate the abundance of H3K9me2 and H3S28p to the promoter regions of CXCL8 (**A**), IL-17 (**B**), TNFA (**C**), IL-6 (**D**), FOS (**E**), and IL-1B (**F**). % input: relative amount of immunoprecipitated DNA compared to input DNA; IgG0, negative control. Bar graphic shows mean ± SEM, * *p* < 0.05, ** *p* < 0.01, *** *p* < 0.001, **** *p* < 0.0001 by using a one-way ANOVA and Bonferroni multiple comparison test. See Supplementary Figure S1 for background control analysis.

The repressive mark H3K9me2 has been reported to be reduced in several inflammatory processes [31,33,49,50], which aligns with our results. In addition, H3S28p is a well-known activation mark associated with stress response and inflammation [31,46,51]. In turn, it has been extensively shown that after burn injury, gene expression changes occur [5,12,18,52], including increases in CXCL8, IL-17, IL-1B, TNFA, IL-6 [5,7,15,16,17,18], and FOS [53,54,55,56]. Interestingly, these genes were also previously reported as epigenetically regulated in pathologies related to inflammatory processes [57,58,59,60,61]. To elucidate the functional implication of the altered epigenetic marks in burn patients, we performed chromatin immunoprecipitation (ChIP) assays to analyze the enrichment of H3K9me2 and H3S28p in the promoter region of selected proinflammatory genes. Interestingly, the repressive mark H3K9me2 stayed significantly down through the first 10 dpb in the CXCL8 and IL-17 promoters and at 5 and 10 dpb in the TNFA promoter, while the abundance of H3S28p on CXCL8, IL-17, and TNFA promoters showed no change in burn patients when compared with healthy controls (Figure 3A–C). In the opposite way, non-changes in H3K9me2 abundance were detected in IL-6, FOS, and IL-1B promoters, but the enrichment of H3S28p was significantly reduced in both IL-6 and FOS gene promoters at all analyzed times (Figure 3D,E). The IL-1B promoter showed a decrease in the abundance of H3S28p only at 5 and 10 dpb (Figure 3F). The above strongly suggests that histone PTMs contribute to the tight regulation of inflammatory genes after burn injury. Likely, a decreased occupancy by H3K9me2 could prevent the repression of inflammatory genes, while the reduction in the enrichment of H3S28p could be routed to prevent an exacerbated increase in inflammatory gene expression.

**Figure 4:**
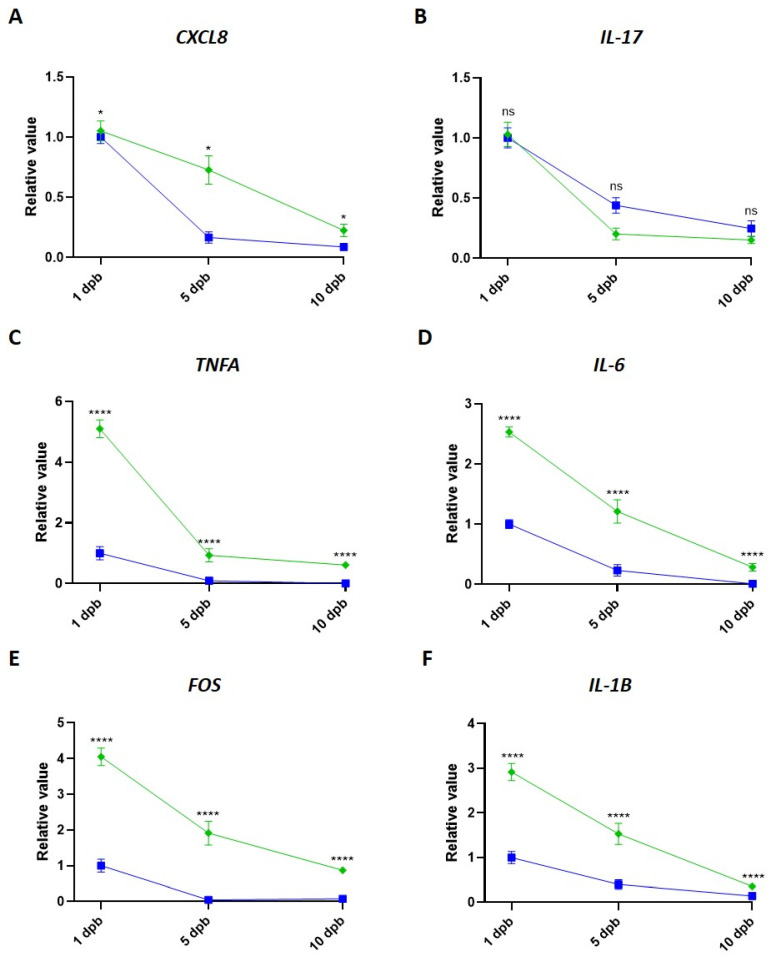
Dynamic of inflammation gene promoter occupancy by H3S28p and H3K9me2 in response to burn injury. ChIP values were used to analyze the relative abundance of H3S28p (green line) and H3K9me2 (blue line) at 1, 5, and 10 dpb in the promoter regions of CXCL8 (**A**), IL-17 (**B**), TNFA (**C**), IL-6 (**D**), FOS (**E**), and IL-1B (**F**) genes. The promoter enrichment value was normalized to H3K9me2 at 1 dpb. Bar graphic shows mean ± SEM, * *p* < 0.05, **** *p* < 0.0001, ns = nonsignificant by using a two-way ANOVA and Bonferroni multiple comparison test.

We then compare the dynamic of gene promoter occupancy by the H3K9me2 and H3S28p throughout time, analyzing their abundance at 5 dpb and 10 dpb relative to occupancy at 1 dpb. Our results showed that occupancy by both epigenetic marks decreased over time for all the studied promoters (Figure 4). Interestingly, the occupancy of the activation mark H3S28p showed significantly higher levels in the CXCL8, TNFA, IL-6, FOS, and IL-1B promoters than the repressive mark H3K9me2 at 1, 5, and 10 dpb (Figure 4A,C–F). It is also noteworthy that H3S28p occupancy decreases faster after 1 dpb in TNFA, IL-6, FOS, and IL-1B than H3K9me2, which could reveal the importance of this mark in the attempt to control the inflammatory gene expression during the response to burn injury.

### 3.4. Sepsis Development of Severe Burn Patients Is Preceded by Differential H3K9me2 and H3S28p Recruitment to Inflammatory Gene Promoters

**Figure 5:**
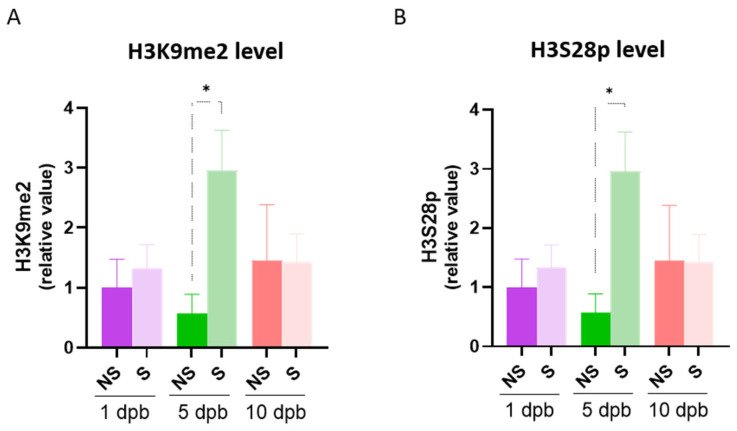
H3K9me2 and H3S28p increased levels at 5 dpb in patients that developed sepsis. Levels of H3K9me2 (**A**) and H3S28p (**B**) were evaluated at 1, 5, and 10 dpb in 8 burn patients that developed sepsis (S) and 4 burn patients who did not develop sepsis (NS). Bar graphic shows mean ± SEM, * *p* < 0.05 by using a one-way ANOVA and Bonferroni multiple comparison test.

As sepsis is a common complication of burns, we were prompted to analyze the levels of PTMs in septic and non-septic patients. As we can see in Figure 5, the levels of H3K9me2 and H3S28p at 5 dpb were significantly increased in patients who developed sepsis compared to patients who did not develop sepsis. No changes were detected in H3K27me1 and H3K4me3. The above points to changes in levels of these H3 PTMs potentially having a role in sepsis development.

**Figure 6:**
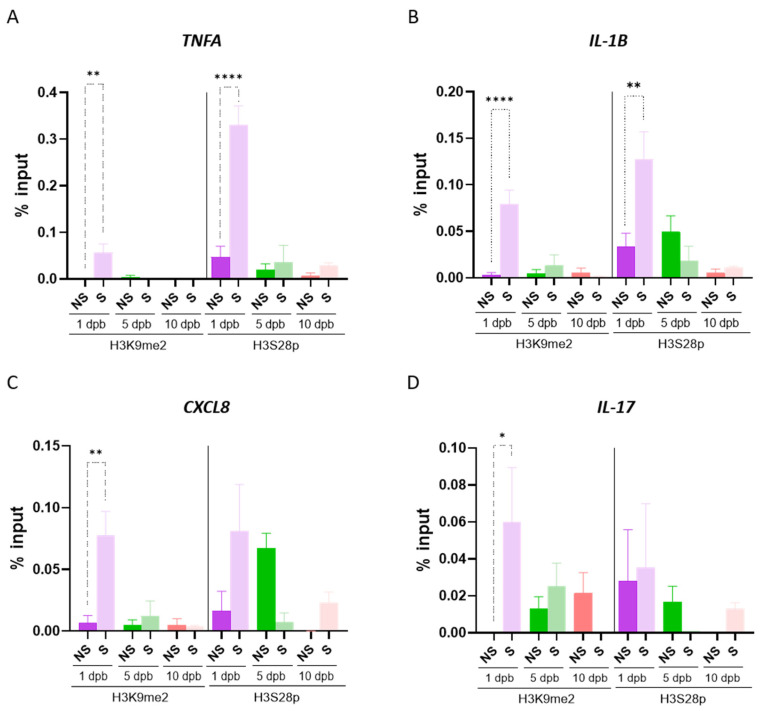
H3K9me2 and H3S28p are differentially recruited to inflammatory gene promoters in septic and non-septic burn patients. Chromatin immunoprecipitation assays were performed in PMBCs of septic and non-septic burn patients at 1, 5, and 10 dpb. Levels of recruitment of H3K9me2 and H3S28p to the promoter regions of TNFA (**A**), IL-1B (**B**), CXCL8 (**C**), and IL-17 (**D**) were evaluated. % input, relative amount of immunoprecipitated DNA compared to input DNA. Bar graphic shows mean ± SEM, * *p* < 0.05, ** *p* < 0.01, **** *p* < 0.0001, by using a one-way ANOVA and Bonferroni multiple comparison test.

Afterward, to ascertain the relevance of the altered levels of H3K9me2 and H3S28p in patients who developed sepsis, we performed ChIP analysis at 1, 5, and 10 dpb. Interestingly, the repressive mark H3K9me2 was significantly enriched in *TNFA*, *IL-1B*, *CXCL8*, and *IL-17* gene promoters at 1 dpb in patients who developed sepsis (Figure 6A–D). Likewise, the occupancy by H3S28p was increased in *TNFA* and *IL-1B* promoters at 1 dpb in patients who developed sepsis (Figure 6A,B). No changes were observed in IL-6 and FOS gene promoter recruitment, neither for H3K9me3 nor for H3S28p (Supplementary Figure S3). Overall, these data indicate that epigenetic changes arise before sepsis establishment, suggesting that they may contribute to an imprecise control of inflammatory genes and, consequently, to sepsis development.

## 4. Discussion

Burn injury has been shown to activate extensive gene expression changes [10,21,55,62,63]. The epigenetic components are essential regulators of inflammation-associated genes [23,24,25,26,27,28,64] and, in burn injury, regulatory non-coding RNAs, DNA methylation, and histone acetylation have been implicated in local [41,65,66,67] and systemic responses [34,35,37]. A recent work described that transcriptomic expression patterns associated with specific epigenetic signatures in circulating immune cells could predict burn patient outcomes [68]. A significant increase of H3K4me1, H3K4me3, and H3S10p in spinal neurons has been identified in a murine burn injury model, contributing to pain perception regulation [39]. Additionally, timely and spatial regulation of H3K27, H4K5, H4K8, and H4K12 acetylation was a crucial factor in the local burn healing process in a porcine burn model [40]. Even though it has been described that epigenetic factors are involved in burn injury response, our understanding of the role of PTMs of histone H3 remains limited, and the epigenetic systemic response in the PBMCs of severe burn patients remains to be elucidated. In this work, we describe for the first time changes in H3 PTMs and the enrichment of specific H3 marks on several inflammation-related genes in circulating leukocytes in response to severe burn injury.

First, we describe eight PTMs of histone H3 with altered levels at 5 dpb (Figure 1), and at least four of them (H3K4me3, H3K9me2, H3K27me1, and H3S28p) also at 1 and 10 dpb (Figure 2). Unexpectedly, we found that the level of these four marks was diminished in the three times evaluated, despite their opposite individual impact on transcription regulation. The above lets us ask about the significance of these alterations over the regulation of genes involved in inflammatory responses.

**Figure 7:**
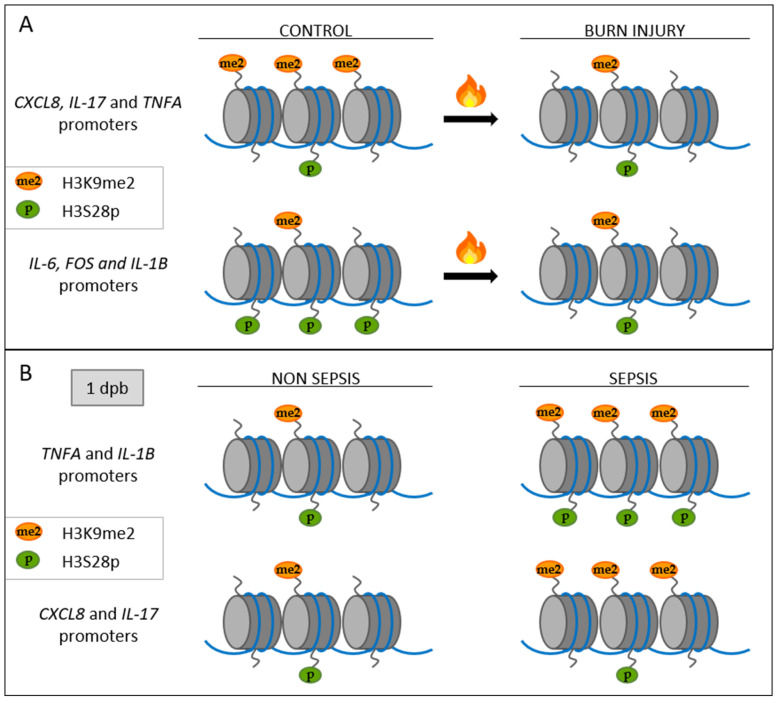
Differential recruitment of H3K9me2 and H3S28p to inflammation gene promoters in response to severe burns (**A**) and non-septic and septic burn patients (**B**).

In murine burn models, an expression rise of several inflammatory mediators, including IL-1B, IL-6, TNFA, and IL-17, has been reported [18,52]. Likewise, in severe burn patients, it an increase of the serum interleukins IL-8, IL-17, IL-1B, TNFA, and IL-6 from a few hours to 21 days post-burn both in adults [6,7,15,52] and in pediatric patients [5,18] has been described. Consequently, the expression of other proteins as the transcription factor c-fos is also induced [53,54,55,56]. Remarkably, transcriptional upregulation of IL-8, IL-17, IL-1B, TNFA, IL-6, and FOS genes has been detected in PBMCs in murine burn models for up to 14 dpb [10,21,55] and in severe burn patients at 3 and 7 dpb [62,63]. Interestingly, these genes have been previously described as potential targets of epigenetic regulation by histone H3 PTMs [57,58,59,60,61]. Particularly, H3K9me2 has been reported as an epigenetic regulator of TNFα, IL-6, and IL-1β in the human-derived liposarcoma cell line SW872 stimulated with LPS [57]. H3K9me2 regulates IL-17 in a murine T cell transfer model of colitis [58], IL-6 in an inflammatory vascular smooth muscle cell model [59], Il-8 in an inflammatory intestinal epithelial cell model [60], and c-FOS in an aged-related impaired memory neuronal cell model [61]. In this study, we demonstrated a reduced enrichment of the repressive mark H3K9me2 in the promoter region of the inflammatory genes CXCL8, IL-17, and TNFA early after the burn (Figure 3 and Figure 7A). According to this, the levels of H3K9me2 decrease in inflammatory processes, reducing the repression of inflammatory genes [59,60]. Previously, it was described that the repressive mark H3K9me2 diminished in some vascular inflammation models, correlating with the increase of transcription factors that activate inflammation-responsive genes [59]. Likewise, in HT-29 cells stimulated with lipopolysaccharide (LPS), it was shown that CXCL8 induction was triggered by a decrease of H3K9me2 and an increase of H3 acetylation at the promoter region [60]. In contrast, in other inflammatory models, such as LPS-induced macrophage cell lines, non-changes in the enrichment of H3K9me2 in IL-6 and TNFA promoters were observed, and the changes in the level of these inflammatory proteins are due to an increase of activation marks [29]. In our study, we observe an evident diminution of the enrichment of H3K9me2 in three of the six inflammation-associated genes analyzed, which suggests that after severe burn injury, H3K9me2 could play a key role in regulating the inflammatory response. This could be a mechanism to counteract the overexpression of these inflammatory genes after burns.

On the other hand, we found a reduction in the enrichment of the activation mark H3S28p in the IL-6, FOS, and IL-1B promoters (where no change was observed for the H3K9me2 repressive mark) (Figure 3 and Figure 7A). H3S28p is a well-known activation mark associated with stress and inflammation [31,47,51]. Previously, it has been described that H3S28p participates in the displacement of the Polycomb repressive complex and, in conjunction with the switch of methylation to acetylation in the H3K27 residue, leads to gene transcription activation [46,69]. Additionally, H3S28p functions as a recognition signal for some proteins, such as 14-3-3 family members, which in turn direct the recruitment of chromatin-modifying proteins, such as HATs [70,71], and reduce the binding of others, such as HDACs, leading to histone acetylation and transcriptional induction [47]. Interestingly, the epigenetic mark H3S28p was present in almost 50% of up-regulated genes in a stress-induced 3T3 fibroblast model [47]. Furthermore, H3S28p was augmented in mouse macrophages stimulated with LPS at 30 and 60 min and enriched in induced genes, such as INFB1, IL-27, CXC110, TNF, and IL-12, which suggests their involvement in the regulation of inflammatory process [51]. Unexpectedly, our study revealed a decreased occupancy of H3S28p in the inflammatory gene promoters of IL-6, IL-1B, and FOS, contrasting with the reported overexpression of these genes in response to burns. In a similar way, a previous study using monocytes of patients with severe coronary atherosclerosis stimulated with LPS reported an increased level of TNFα associated with diminished levels of the repressive mark H3K27me3, but also with decreased levels of the activation mark H3K4me3 in the TNFA promoter [72]. According to our data, we suggest that the regulation in the levels of the activation mark H3S28p could be a mechanism to prevent an exacerbated increase in the inflammatory gene expression, which is in part mediated by the decreased occupancy of the repressive mark H3K9me2 in the inflammatory gene promoters. Taken together, our results suggest that inflammation in response to severe burn injury involves epigenetic changes oriented to modulate the exacerbated expression of proinflammatory genes.

One of the most common and serious complications of severe burn injury is the development of burn wound infections, which may result in sepsis in cases where the host response is highly dysregulated [42,73,74]. The incidence of sepsis in severe burn injury ranges between 3 and 30%, and more than half of burn-related deaths are due to septic shock and multiple dysfunction organ syndrome (MODS) [75,76]. An efficient and early diagnosis of sepsis is a critical step to improve the outcome of burn patients. Several biochemical and molecular sepsis biomarkers are currently used [73,77,78,79]; however, the discovery of better and earlier sepsis biomarkers in a burn context is still a need. In this study, we found that levels of H3K9me2 and H3S28p were increased from day 5 post-burn in patients who developed sepsis. In fact, enrichment of the repressive mark H3K9me2 was increased in promoters of TNFA, IL-1B, CXCL8, and IL-17 at 1 dpb in patients that develop sepsis. Similarly, H3S28p was found to be more enriched in TNFA and IL-1B promoters at 1 dpb in patients that developed sepsis (Figure 6 and Figure 7B). Due to burn patients included in this study developing sepsis between day 5 and 11 dpb, we hypothesize that early alterations of histone PTMs (levels and/or promotor occupancy) could have an impact on the regulation of these inflammatory genes. Future studies are needed to know whether H3K9me2 and H3S28p marks may predispose burn patients to sepsis. Likewise, studies analyzing the levels of epigenetic writers and erasers should be conducted to understand the molecular mechanism that leads to the epigenetic alterations observed in this study. Epigenetic control depends on the recruitment of distinctive marks in a precise moment and cellular context; thus, extending our study to other PTMs using genome-wide approaches is necessary to obtain a better picture of the inflammation regulation after severe burns. This would eventually lead us to propose new possible therapeutic strategies directed to control the misregulation of gene expression in burn injury responses.

There are some limitations in this study that could be addressed in future research. The modest sample size could lead to an underpowered analysis and, in fact, prevented the possibility of analyzing the impact of factors such as burn etiology and age of patients over the epigenetic alterations. The lack of analysis of variables such as gender, individual medication, and baseline inflammation status represents a risk of bias in this study. Additionally, ChIP analysis in bulk PBMC made it impossible to differentiate the epigenetic state in specific cell types, which should be overcome to clarify the role of cell subtypes in the global inflammatory state after severe burns.

## 5. Conclusions

We described systemic alterations in epigenetic marks of histone H3 in response to severe burn injury. Our data suggest that inflammation after severe burns may be regulated by differential enrichment at inflammation gene promoters of both repressive H3K9me2 and activating H3S28P marks. In addition, epigenetic misregulation of H3 PTMs may be involved in the development of critical complications such as sepsis in severe burn patients.

## Figures and Tables

**Table 1:** Characteristics of burn patients and healthy controls.

	Patient (N = 12)	Controls (N = 6)	*p*-Value
Age in years (range)	30.83 (15–81)	33.67 (15–71)	0.5681
Etiology (Fire/Electricity)	7/5	-	-
% TBSA (range)	36.92 (15–60)	-	-
Sepsis	8	-	-
IL-6 (pg/mL)	913.4 (1 dpb)	<3 pg/mL	*
	901.2 (5 dpb)		*
	715.2 (10 dpb)		**

Data are shown as mean ± SEM, * *p* < 0.05, ** *p* < 0.001 by using the Mann–Whitney test for age analysis and the Kruskal–Wallis test for IL-6 analysis; dpb: days post-burn.

## Data Availability

No new data were created or analyzed in this study. Data sharing is not applicable to this article.

## Supplementary Materials

The following supporting information can be downloaded at: https://www.mdpi.com/article/10.3390/life14121581/s1. Supplementary Figure S1: Background control analysis for chromatin immunoprecipitation assays. Irrelevant antibody was used as background control for chromatin immunoprecipitation assays. Statistical analysis shows a significant difference for IL-6, IL-1b, TNFa, IL-17, IL-8, and cFos both using H3S28p as H3K9me2 antibodies, except for IL-6 vs. H3K9me3, where no differences were obtained. % input, relative amount of immunoprecipitated DNA compared to input DNA; IgG, negative control. Bar graphic shows mean ± SEM, * *p* < 0.05, ** *p* < 0.01, *** *p* < 0.001 by using an unpaired *t*-test.; Supplementary Figure S2: List of PTMs of histone H3 included in the multiplex analysis. Supplementary Figure S3: H3K9me2 and H3S28p recruitment to inflammatory gene promoters IL-6 and FOS in septic burn patients. Chromatin immunoprecipitation assays were performed in PMBCs of septic and non-septic burn patients at 1, 5, and 10 dpb. Levels of recruitment of H3K9me2 and H3S28p to the promoter regions of IL-6 (A) and FOS (B) were evaluated. % input, relative amount of immuno-precipitated DNA compared to input DNA. Bar graphic shows mean ± SEM. Data were analyzed by using a one-way ANOVA and Bonferroni multiple comparison test.
