# Supplementary figures and images for: Enrichment of H3S28p and H3K9me2 Epigenetic Marks on Inflammatory-Associated Gene Promoters in Response to Severe Burn Injury
Source: Life (Basel). 2024 Dec 1;14(12):1581. doi: 10.3390/life14121581 (PMC11677237; doi:10.3390/life14121581)

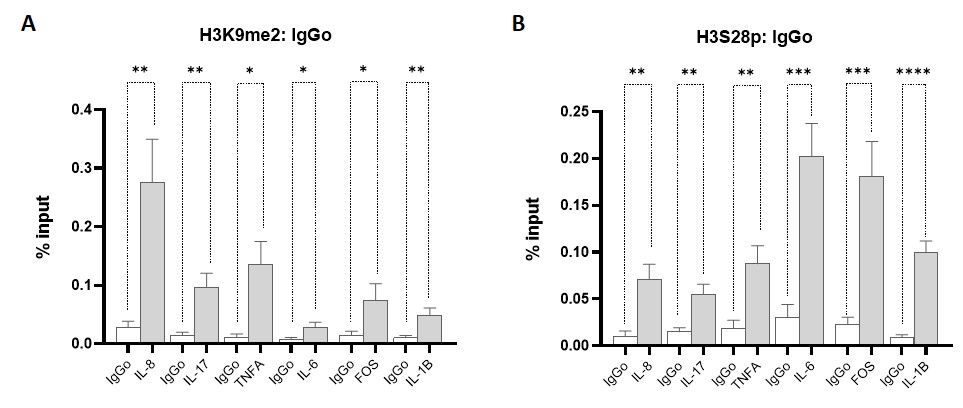

Supplement: Supplementary file 1 [file life-14-01581-s001.zip › Supplementary Figure S1.jpg]

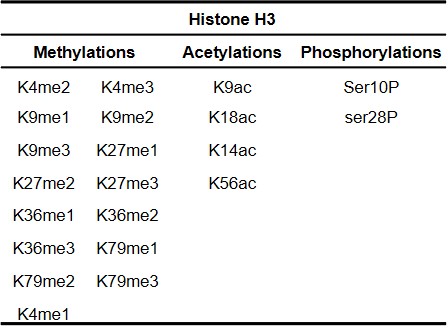

Supplement: Supplementary file 1 [file life-14-01581-s001.zip › Supplementary Figure S2.jpg]

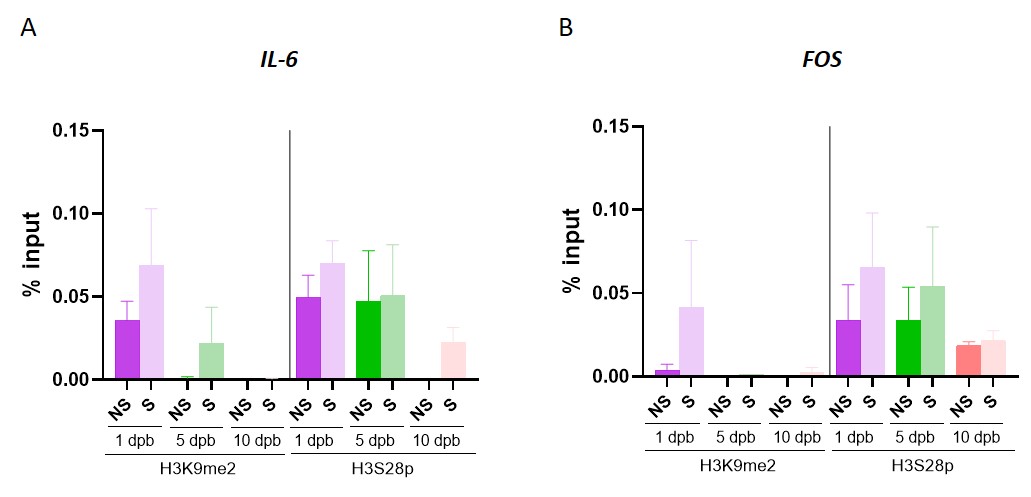

Supplement: Supplementary file 1 [file life-14-01581-s001.zip › Supplementary Figure S3.jpg]
